# Transcription Regulatory Sequences in Coronaviruses: Mechanistic Foundations for Antivirals and Vaccines

**DOI:** 10.4014/jmb.2609.09039

**Published:** 2026-10-19

**Authors:** Jinjong Myoung

**Affiliations:** Korea Zoonosis Research Institute, Department of Bioactive Material Science and Genetic Engineering Research Institute, Jeonbuk National University, Jeonju 54531, Republic of Korea

**Keywords:** SARS-CoV-2, Coronavirus, Transcription regulatory sequence, TRS-L, TRS-B, Discontinuous transcription, Subgenomic RNA, RNA structure, Replication-transcription complex

## Abstract

Coronaviruses employ a distinctive discontinuous transcription mechanism to generate a nested set of subgenomic mRNAs (sgRNAs) from a single large positive-strand RNA genome. Transcription regulatory sequences (TRSs)-comprising a leader TRS (TRS-L) near the 5′ end and multiple body TRSs (TRS-Bs) upstream of downstream open reading frames-serve as the primary *cis*-acting signals that direct polymerase pausing and template switching during negative-strand synthesis. The COVID-19 pandemic spurred intensive investigation of SARS-CoV-2 gene expression, yielding high-resolution transcriptome maps, genome-wide RNA structure data, and detailed structural models of the replication-transcription complex (RTC). TRS function is therefore best understood as a multi-scale property determined not by the core motif alone, but by the integration of primary-sequence complementarity, local RNA secondary structure, long-range RNA–RNA interactions, and RTC dynamics. This review provides an integrated view of TRS structure–function relationships, with emphasis on how these features collectively shape sgRNA abundance, junction diversity, recombination potential, and prospects for diagnostic and antiviral applications, including vaccines and therapeutics.

## Introduction

The emergence of SARS-CoV-2 in late 2019 precipitated not only a global health crisis but also a scientific mobilization without precedent in virology [1-3]. Coronavirus gene expression has been a major focus of research, both because the mechanisms are unusual and because they have practical value for diagnostics and therapy [4-7]. Coronaviruses, members of the order Nidovirales, utilize a distinctive transcription mechanism among RNA viruses, characterized by discontinuous transcription that generates a nested set of subgenomic RNAs (Table 1), each bearing a common 5′ leader sequence fused to distinct downstream body regions [4, 5, 8-10]. TRSs, which signal and govern this process, have long been recognized as critical cis-acting elements within the genomes of viruses in the order Nidovirales [10-13]; however, their precise mechanism of action remained incompletely elucidated [9, 14].

The COVID-19 pandemic catalyzed intensive investigation of coronavirus gene expression [15-20] and substantially advanced mechanistic understanding of discontinuous transcription. Transcriptome maps generated by both short-read and direct RNA nanopore sequencing revealed that sgRNA production is a regulated, hierarchical output of the RTC whose junction composition varies with cell type, infection stage [5, 21, 22], and viral lineage [23]. These findings elevated TRSs from mere annotation features to *bona fide* regulatory elements whose sequence and structural context encode information read by the viral polymerase during RNA synthesis [5, 9, 14]. At the same time, structural biology produced detailed atomic models of the SARS-CoV-2 replication–transcription complex (RTC), while genome-wide RNA structure probing and interactome mapping revealed a surprisingly complex set of RNA structures around TRS sites [24-33]. Taken together, these advances motivate the present review, which aims to map TRS structure–function relationships and identify critical gaps in our understanding.

### What Makes Coronavirus Transcription Unique?

Coronaviruses replicate within membrane-associated compartments, utilizing a multi-protein RTC whose core polymerase activity resides in nsp12 (RNA-dependent RNA polymerase, RdRp) together with essential cofactors nsp7 and nsp8 [4, 24, 31-33]. Unlike many RNA viruses that produce subgenomic transcripts through internal initiation or subgenomic promoter recognition, coronaviruses generate their mRNAs by a discontinuous mechanism (Fig. 1): during negative-strand synthesis, the polymerase is proposed to pause or decelerate at a TRS-B site, allowing template switching to the TRS-L near the genome’s 5′ end, producing a negative-sense template that, upon copying, yields a leader-containing sgRNA [4, 5, 9, 34-38]. This process is distinct from DNA-dependent transcription systems and is executed entirely by the viral RNA-dependent RNA polymerase on an RNA template [4, 9].

The result is a set of mRNAs that all share the same 3′ end and all carry the 5′ leader sequence. Their relative amounts depend on how efficiently template switching occurs at each TRS-B, so the genome itself encodes a program of gene expression (Fig. 1). During infection, the virus reshapes host membranes to form double-membrane vesicles (DMVs), which enclose the RTC and the viral RNA in a separate compartment (Fig. 1), further specializing in the physical environment in which discontinuous transcription occurs [39-41].

### Defining TRSs: TRS-L, TRS-B, and Leader–Body Junctions

In the standard model, TRSs fall into two classes: a single TRS-L within the 5′ leader, and a set of TRS-Bs, each located just upstream of a downstream ORF [5, 9, 10, 14]. TRS-L and TRS-B share a conserved 6-7 nucleotide core sequence (CS), typically ACGAAC in SARS-CoV-2, surrounded by variable flanking sequences that together determine switching efficiency (Fig. 3A) [5, 10, 14, 42-47]. During negative-strand synthesis, the nascent RNA complementary to a TRS-B can base-pair with the TRS-L region (Fig. 1B), enabling the polymerase to relocate to the leader template and thereby fuse leader and body sequences in the resulting sgRNA [4, 5, 9, 37].

Transcriptome profiling showed that sgRNAs occur in a consistent order of abundance, with the nucleocapsid (N) mRNA the most abundant. The same work also uncovered a wide range of non-canonical junctions that do not match any annotated TRS-B [5, 21, 22]. This observation carries an important mechanistic implication: TRS function is inherently probabilistic. TRS features influence how likely template switching is, rather than acting as simple on/off switches. High-resolution mapping, based on counting junction-spanning reads and assembling transcripts with graph-based methods, showed that canonical and non-canonical leader-body fusions coexist. This pattern reflects stochastic polymerase behavior, shaped by sequence complementarity and by the surrounding RNA structure [5, 48].

### A Multi-Layered View of TRS Structure

**Primary sequence: core motif and flanking context.** At the primary sequence level, TRS function depends on complementarity between the nascent negative-strand RNA and the leader region: base-pairing between these sequences facilitates the template switch that produces leader-containing transcripts [4, 5, 9, 14, 49]. While the CS comprises only 6−7 nucleotides, its flanking context plays a substantial role in determining junction efficiency (Fig. 2A). In SARS-CoV-2 this is readily appreciated from a direct alignment of the leader and body TRSs (Fig. 3A). All nine canonical TRS-Bs carry an identical ACGAAC core, yet the length of uninterrupted identity with the leader context by a factor of two, from 12 nt at the M site to the bare 6-nt core at ORF6; the E and ORF6 sites are the only two that lack the AAACGAAC extension shared by the remaining six. Because the thermodynamic cost of forming the TRS-L: cTRS-B duplex scales with the length and composition of this complementary tract (Fig. 3B), the same core motif is presented to the RTC in substantially different energetic contexts at different positions in the genome. In particular, AU-rich or otherwise structurally permissive flanking regions, documented in several experimentally studied systems, affect RNA folding and duplex stability near the switch point, thereby modulating overall efficiency [14, 46, 47]. Systematic analyses showed that sequences matching the TRS-L core sequence (CS) occur more often at sgRNA junction sites than elsewhere in the genome. Junctions were also detected at sites with only partial matches, indicating that template switching does not require perfect base pairing but instead occurs with a probability that depends on the degree of complementarity [14, 46]. Mutagenesis studies in transmissible gastroenteritis virus and SARS-CoV demonstrated that flanking sequence alterations shift the balance of sgRNA production across genes [50], establishing that the TRS ‘signal’ extends beyond the core motif [51] to include adjacent nucleotides affecting RTC pausing and nascent RNA conformation [9, 46, 47]. Crucially, early reverse genetics studies utilizing Transmissible Gastroenteritis Virus (TGEV) expression vectors demonstrated that artificial expansion or optimization of the core and flanking sequence complementarity could precisely modulate the transcriptional efficiency of heterologous genes, proving that TRS-B operates as a tunable promoter rather than a simple binary switch [47, 52-54]. In addition, classical mutagenesis and defective interfering (DI)/minigenome studies in MHV and TGEV indicated that sgRNA synthesis efficiency is largely determined by the stability of the TRS-L/cTRS-B duplex. The TRS core provides the primary complementarity, whereas flanking sequences further modulate efficiency both by extending the duplex and by altering the local accessibility of the TRS-B [36, 37, 46, 47, 49, 52, 55], strongly suggesting that the free energy (ΔG) of duplex formation is a major determinant of the probabilistic efficiency of this process (Fig. 3B).

**Local RNA Secondary Structure near TRSs.** Chemical probing methods applied to infected cells reveal the secondary structure of the SARS-CoV-2 genome at nucleotide resolution. The results show that much of the genome is folded into structured elements, and that regions around TRS sites are no exception [25-27, 46]. SHAPE (selective 2′-hydroxyl acylation analyzed by primer extension) and its in-cell variant icSHAPE produce reactivity profiles at single-nucleotide resolution. These profiles identified structured elements throughout the genome, including the 5′ leader and the regions surrounding body TRSs [25-27, 56]. These data suggest that TRS accessibility is dynamic [57]: local folding may expose or hide the core motif, and in doing so alter the probability that the leader and body sequences pair during template switching.

Structural heterogeneity is the rule rather than the exception. Several folded states coexist in equilibrium, and switching efficiency probably depends on the fraction of molecules in which the TRS core is accessible for base pairing [26, 27]. In several coronaviruses, structured elements near TRS sites are conserved, suggesting functional constraints that balance replication, transcription, and other RNA-level processes such as protein binding and translational control [25-27, 58]. For example, stem-loop 3 (SL3) in SARS-CoV folds into a stable hairpin, with the TRS-L sequence positioned in the 3′ half of the stem; NMR spectroscopy confirmed this arrangement [46]. SARS-CoV-2 differs. Its SL3 does not settle into a single conformation but interconverts between folded and partly open states. This flexibility may be what allows TRS-L to become available for pairing with TRS-B sequences when the polymerase switches templates [59]. Bovine coronavirus (BCoV) similarly contains SL3 in the hairpin loop region [46], whereas Mouse hepatitis virus (MHV) presents an unstable SL3 structure at 37°C, potentially enabling rapid structural transitions [60]. MERS-CoV notably lacks SL3 altogether, positioning TRS-L as single-stranded and employing alternative regulatory mechanisms [61, 62] (Table 2). It should be emphasized that these structures are not known to equivalent standards. The SARS-CoV SL3 has been determined experimentally, whereas the SARS-CoV-2 element is best described as a conformational ensemble inferred from in-cell probing and comparative modelling, and the published probing datasets differ in the base-pairing they assign to this region. The attractive inference, that a structural difference at SL3 accounts for a difference in template-switching behavior between the two viruses, has not been tested directly, since no study has yet manipulated SL3 conformation in SARS-CoV-2 and measured the consequence for junction usage. The comparison in Table 2 should therefore be read as a map of what is and is not known, rather than as a mechanistic account. Additionally, genome-wide mapping reveals that seven of nine TRS-B sites in SARS-CoV-2 reside within stem-loop structures with differential exposure of core sequences, suggesting that RNA structural contexts fine-tune the kinetics and efficiency of discontinuous RNA synthesis [14].

**Long-range RNA interactions and three-dimensional organization.** Sequencing of Psoralen crosslinked, Ligated, And Selected Hybrids (SPLASH) and related crosslinking-ligation methods help identify the RNA-RNA interactome. These methods use a chemical crosslinker to trap RNA duplexes inside cells. The paired fragments are then ligated together and sequenced, so each read reports two regions that were in contact. Applied to SARS-CoV-2, these methods identified numerous contacts between distant genomic regions and between regions within individual sgRNAs. Some of these contacts involved sequences lying close together in the genome; others spanned tens of kilobases. Notably, contacts were detected between the leader and body regions, bringing sequences that are far apart in linear sequence into direct spatial contact [14, 28, 29]. These contacts suggest that the leader engages multiple genomic sites, potentially pre-organizing conformational networks that influence which TRS-Bs are kinetically accessible during template switching. Work on the architecture of the SARS-CoV-2 RNA genome within the virion revealed that the RNA adopts a defined tertiary organization inside the nucleocapsid, raising the possibility that analogous higher-order structuring within replication compartments may constrain TRS exposure and polymerase scanning dynamics [63].

Taken together, these multi-scale features, including sequence motif, local secondary structure, and long-range genome organization, suggest that TRS function arises from their combination rather than from any one of them alone. Predictive understanding will require integration across all these levels [25-30, 63].

**Protein context: the nucleocapsid protein as a trans-acting modulator.** TRS function is not determined by RNA alone. The nucleocapsid (N) protein is the most abundant viral RNA-binding protein in infected cells and acts as an RNA chaperone that promotes template switching: in TGEV, N is required for efficient subgenomic RNA synthesis and facilitates annealing of the nascent cTRS-B to TRS-L, and chaperone-deficient N mutants are impaired selectively in transcription rather than in replication [64]. Structural analysis of the SARS-CoV-2 N C-terminal domain indicates sequence-selective recognition of TRS-containing RNA with a contribution from the flanking region, which offers a molecular rationale for why an invariant core motif can behave differently in different sequence contexts. N also undergoes RNA-dependent liquid–liquid phase separation and is recruited to replication organelles, so that locally high N concentrations could lower the kinetic barrier to duplex formation, remodel inhibitory structures around a TRS-B, or conversely sequester TRS elements and suppress switching. Consistent with a functional link, the R203K/G204R substitutions, which lie in the N linker region and simultaneously create a TRS-like motif in the N gene, are associated with an additional subgenomic RNA and with altered sgRNA abundance in the Alpha lineage [23, 65].

How N exerts these effects is unresolved. It may act as a general RNA chaperone, as a factor that recognizes individual TRS sites, or simply by concentrating the reactants within replication organelles - and these possibilities are not mutually exclusive. This uncertainty marks one of the clearest gaps between the RNA-centric framework developed above and the protein environment in which template switching actually occurs.

### Mechanistic Model of Discontinuous Transcription

The prevailing model proposes that during negative-strand synthesis the RTC pauses or slows at a TRS-B. This allows the 3′ end of the nascent negative strand, which now carries the complement of the TRS-B, to base-pair with TRS-L in the leader. Synthesis then resumes using the leader as template [4, 5, 9, 37]. Switching probability at each TRS-B is jointly determined by core complementarity, local RNA folding stability, and RTC kinetics, producing gene-specific differences in sgRNA yield [5, 9, 24, 36]. Together with earlier models of coronavirus discontinuous transcription [34, 66], recent data are consistent with a pause-and-switch framework, first proposed by Sawicki and Sawicki [34, 66] and later refined in TGEV by Enjuanes and colleagues [9, 49], in which slowing of the RTC near a TRS-B allows the nascent negative strand to base-pair with TRS-L [37].

Structural and functional analyses of the RTC underscore that nsp12 operates in concert with cofactors and accessory enzymes whose coordinated activities affect processivity, pausing, and fidelity, implying that TRS behavior is inseparable from RTC organization [24, 31-33, 67, 68]. Template switching during transcription is not a passive process; it requires active participation by various viral replicase proteins. The replication-transcription complex (RTC) must pause at a body transcription regulatory sequence (TRS-B), release the 3′ end of the nascent strand from its template, and resume synthesis at the leader TRS (TRS-L) roughly 20 kb upstream. Sequence complementarity between TRS-B and TRS-L is therefore necessary but not sufficient: the switch is a mechanical event, dependent on helicase activity and shaped by the interplay between RTC components and local RNA structure. This helicase, nsp13, is the viral superfamily-1 helicase. Two copies are bound per RTC; nsp13 translocates 5′→3′ on single-stranded RNA and unwinds duplex RNA ahead of nsp12, and because one copy engages the template strand with a polarity opposite to that of polymerase translocation, its ATP-dependent activity governs both the speed of forward elongation and the capacity of the complex to move backwards. It is in this second role that nsp13 is directly implicated in template switching. Single-molecule magnetic tweezers experiments, which follow individual coronavirus RTCs in real time, have provided direct evidence for this requirement [69]. During uninterrupted elongation, this activity keeps nsp12 in a fast nucleotide addition (FNA) mode. When the RTC meets conditions that slow incorporation (for example, reduced NTP availability or stable secondary structure flanking a TRS-B), nsp13 disengages, and the polymerase enters the slower catalytic pathways designated SNA and VSNA (slow and very slow nucleotide addition). This kinetic deceleration is proposed to constitute the initial pausing event at a TRS. The second helicase copy of nsp13 translocates on the template strand with opposite polarity to the polymerase and is therefore expected to push the RTC backward, a process termed backtracking [69]. Crucially, this backtracking is not diffusive but processive, spanning tens to hundreds of nucleotides, and it absolutely requires nsp13 ATPase activity; an ATPase-dead mutant (K288A) completely abolishes all reversal events. As the RTC reverses, the displaced 3' end of the nascent minus-strand RNA snaps back and self-anneals, enabling intramolecular template switching followed by copy-back RNA synthesis. The probability of these reversals increases with the thermodynamic stability of the downstream duplex RNA fork and decreases when NTP concentrations are saturating, consistent with the idea that TRS-flanking structures act as kinetic barriers that bias the RTC toward pausing and backtracking. An important detail is that the two copies of nsp13 cannot translocate simultaneously; they allosterically inhibit one another, ensuring that the transition from forward synthesis to backtracking occurs in a controlled, stepwise manner [69]. The helicase also shows pronounced substrate preference, achieving maximum unwinding velocity with ATP (~328 bp/s, K_m_ ≈ 37 μM) compared to pyrimidines (~147 bp/s for CTP), suggesting that local ATP depletion by the helicase itself may further potentiate pausing at structured TRS regions. This interpretation is supported by the high sequence conservation of nsp13: the orthologs of SARS-CoV, MERS-CoV, and SARS-CoV-2 are closely related, and the structural elements that mediate nsp12-nsp13 interaction appear to be common across coronaviruses [70]. Conservation of this kind is consistent with, though not itself evidence for, a shared pausing-backtracking-switching mechanism among beta-coronaviruses. Meanwhile, the proofreading exoribonuclease nsp14, activated by its cofactor nsp10, corrects errors introduced during the mechanically demanding backtracking phase; disruption of the nsp14-nsp10 interface in MHV impairs both exonuclease activity and viral replication [71]. Altogether, these data establish that TRS-mediated template switching is an enzymatically driven process in which local RNA structure triggers RTC pausing, nsp13 helicase executes backtracking through ATP-powered strand unwinding, and the polymerase completes a controlled strand transfer: a mechanism embedded in the very architecture of the RTC rather than arising from passive thermodynamic equilibrium between TRS sequences.

The extensive non-canonical junctions observed in transcriptome studies further indicate that switching is not confined to canonical TRS sites; RNA structure and polymerase kinetics create additional, ‘opportunistic’ switching points throughout the genome [5, 21, 48].

### Measuring TRS Function: sgRNA Abundance and Junction Diversity

In practice, TRS function is assessed indirectly, through the abundance of each canonical sgRNA and the distribution of leader–body junctions in sequencing data [5, 21, 22]. Kim *et al*. showed that sgRNA abundance in SARS-CoV-2 varies markedly between genes, and that junction-spanning reads allow both canonical and non-canonical junctions to be counted at scale [5]. Computational tools have since been developed to handle discontinuity explicitly: JUMPER assembles transcripts under a model that allows template switching [48], while leader-TRS fusion site (LeTRS) identifies and quantifies leader-TRS junctions across large sequencing datasets [72]. Both improve the detection of low-abundance junctions relative to standard alignment pipelines.

Comparative sgRNA profiling across SARS-CoV-2 variants and other human coronaviruses revealed that while the canonical sgRNA repertoire is conserved, relative abundances shift with viral lineage, host cell type, and infection stage [5, 21-23]. Non-canonical junctions, defined as leader–body fusions not mapping to annotated TRS-Bs, were found throughout the genome, sometimes at appreciable frequencies [5, 21, 48]. These findings suggest that TRS function is better described in probabilistic than deterministic terms, closer to promoter strength in bacterial genetics than to a binary switch. On this view, switching efficiency at a given TRS should vary measurably with cell type, time post-infection, and viral genotype, and the same TRS should yield reproducibly different junction distributions across these conditions. To test whether this holds, one would need to perturb the system experimentally, changing TRS sequences, NTP levels, or local RNA structure, and measure how junction distributions respond. Simply comparing datasets from different samples is insufficient, since those datasets contain too many uncontrolled variables.

### RNA Structure as a Modulator of Switching Probability

*In vivo* RNA structure mapping provided a mechanistic rationale for why identical core motifs produce different transcriptional outcomes in different folding contexts [25-27, 57]. SHAPE reactivity profiles indicated that TRS cores may reside within stems, loops, or dynamic structural ensembles, where transient exposure is required for base-pairing with leader sequences during the switching step [25-27]. The structural heterogeneity observed across the genome, with many regions adopting more than one folded state, is consistent with a model in which RNA conformation controls access to TRS sites and thereby influences switching efficiency [26, 27].

RNA-RNA interaction mapping showed that leader and body regions engage in direct contacts, bringing sequences that are distant in the genome into physical proximity. These contacts may influence the formation of both canonical and non-canonical junctions [28-30]. Contacts between TRS-L and multiple downstream sites were detected across the genome, consistent with a model in which long-range interactions help align leader and body sequences to facilitate template switching [28-30]. This structural layer aligns with transcriptome evidence that non-canonical junctions are frequent and diverse: RNA conformational landscapes may broaden the set of feasible switching events well beyond the annotated TRS-Bs [5, 21, 28].

Beyond local folding, viral RNA is organized within both virions and replication compartments in ways that constrain or promote particular interactions relevant to transcription [39-41, 63]. The architecture of the SARS-CoV-2 RNA genome within the virion adopts a defined organizational conformation, establishing that higher-order structuring is a genuine property of the viral life cycle rather than an *in vitro* artefact [63]. Although virion organization differs from the intracellular replication environment, where RNA associates with the RTC in DMVs, it motivates models in which RNA structural preferences bias which leader-body pairings are kinetically accessible at particular subcellular locations and times [28-30, 39-41, 63].

### Biological Significance of the sgRNA Program

The nested sgRNA set constitutes the coronavirus strategy for expressing multiple ORFs from a single genome while maintaining a common 5′leader that coordinates translation initiation and mRNA stability across all transcripts [5, 9, 14, 48]. The sgRNA hierarchy is dominated by a few highly abundant species; N mRNA is the most abundant, followed by S and other structural/accessory transcripts, with platform- and study-dependent ordering below N. These data imply that the virus tunes relative production to match structural protein demand and infection timing [5, 21, 22]. Because TRS-mediated switching is probabilistic and context-dependent, this hierarchy is best understood as an emergent property of multiple factors: core complementarity, local RNA folding, RTC kinetics, and long-range RNA organization (Fig. 3A) [5, 9, 25-30]. Notably, the extent of leader complementarity does not by itself rank-order sgRNA abundance: the M site carries the longest contiguous complementarity (12 nt) yet N, with 11 nt, is consistently the most abundant transcript (Fig. 3A). Complementarity therefore sets a permissive range rather than determining output, which is precisely what a multi-scale model predicts. Changes in sgRNA abundance have been reported for several SARS-CoV-2 variants and linked to differences in viral phenotype or replication [23, 65]. In the Alpha variant, for example, a trinucleotide substitution in the nucleocapsid gene creates a new TRS-like motif and generates an additional subgenomic RNA that is absent from earlier lineages. Such cases show that the transcription program is not fixed: single mutations can add or remove sgRNA species [65]. Non-canonical junctions are widespread, suggesting that the transcription program includes both ‘designed’ canonical outputs and a background of alternative products that may be tolerated, selected, or occasionally co-opted [5, 21, 48].

Of note, the template-switching mechanism that generates sgRNAs is also mechanistically related to recombination events that produce deletions, duplications, and insertions—a connection with profound implications for coronavirus evolution [4, 9, 73-76]. Studies of SARS-CoV-2 insertion variants supported a role for template switching as a plausible mechanism generating genomic rearrangements [73]. Because coronavirus RdRp is intrinsically capable of template switching, even small changes in sequence context or RTC dynamics may reshape junction usage, sgRNA output and recombination-mediated genome rearrangement [4, 24, 73]. The coronavirus proofreading exoribonuclease (nsp14-ExoN) provides an additional layer of complexity: ExoN-deficient mutants exhibit altered recombination and genome diversity phenotypes, suggesting that proofreading and template switching are functionally coupled [75, 76]. This linkage underscores an important and underappreciated aspect of TRS biology; TRSs are not solely transcriptional regulators but also represent nodes in a broader network of template-switching processes that contribute to coronavirus adaptability and antigenic variation.

### Applications: Diagnostics, Surveillance, and Antiviral Strategies

Because sgRNAs contain leader-body junctions absent from full-length genomic RNA, junction reads have been explored as potential markers of active viral replication, with applications in clinical diagnostics and epidemiological surveillance [5, 77, 78]. However, sgRNA stability and detection sensitivity vary across platforms, and the relationship between sgRNA detection and replicative activity requires careful interpretation [77, 78]. Genome surveillance and variant monitoring further highlight the importance of understanding template-switching signatures, since switching-linked events generate genomic configurations, such as deletions in spike or ORF8, that alter viral phenotype [73, 79].

On the intervention side, the RTC and its accessory enzymes are already central targets: remdesivir inhibits nsp12, and proofreading inhibitors targeting nsp14-ExoN have emerged as a candidate antiviral target, although clinically validated inhibitors remain less advanced than RdRp inhibitors [24, 32, 80, 81]. Genome-wide structure maps offer candidate targets for this purpose [25-27]: conserved structured elements in the 5′leader and around body TRSs are, in principle, accessible to small molecules or to antisense oligonucleotides that occlude a TRS core. Whether TRSs can be exploited as direct antiviral targets deserves a more explicit appraisal than is usually given. The conceptual attraction is selectivity. Because leader–body fusion is dispensable for genome replication but essential for structural protein expression, an agent that occludes TRS-L or a subset of TRS-Bs could in principle suppress virion production while leaving continuous RNA synthesis intact - a mode of action unavailable to nucleoside analogues, which necessarily inhibit replication and transcription together and carry the attendant liabilities of host polymerase cross-reactivity and mutagenicity. The ACGAAC core is also among the most conserved short motifs across sarbecoviruses, raising the prospect of breadth.

Proof of principle exists, though chiefly at the genetic level. Rewiring of the transcription regulatory network attenuates SARS-CoV and PEDV and yields recombination-resistant genomes, as discussed below [82-84]. Antisense approaches directed at the 5'-terminal leader/TRS region provide the closest pharmacological precedent: peptide-conjugated phosphorodiamidate morpholino oligomers targeting this region inhibited murine hepatitis virus and SARS-CoV in cell culture and in mice, and chemically modified antisense oligonucleotides directed against the SARS-CoV-2 5′ leader have shown antiviral activity in animal models.

However, the practical limitations are substantial, and four deserve emphasis. First, the target is a transient RNA–RNA intermediate: the 3′ end of the nascent negative strand is held within the RTC, which is itself enclosed in a double-membrane vesicle that is topologically closed apart from a molecular pore, so delivery of an oligonucleotide or macromolecule to the compartment in which switching occurs remains unproven. Second, a six-nucleotide core is far too short to confer transcriptome-wide specificity; selectivity would have to come from the flanking context, which is precisely the variable part of the element. Third, because switching is probabilistic and non-canonical junctions are abundant, occluding one TRS is more likely to redistribute sgRNA synthesis than to abolish it, and single-nucleotide changes in the core or its flanks can relocate or restore switching, implying a low genetic barrier to escape. Fourth, no small molecule that binds a TRS-proximal element with therapeutically useful affinity and specificity has been reported, and RNA-targeted small-molecule discovery remains at an early stage generally.

On balance, therefore, RdRp and protease inhibitors remain the validated direct-acting options, and TRS-directed intervention is best regarded for the present as a design principle for live-attenuated vaccines and expression vectors, where the sequence can be altered in cis and the attenuation is heritable, rather than as a near-term therapeutic modality. Its principal translational value may ultimately lie less in blocking transcription than in predicting which engineered TRS configurations are genetically stable. Furthermore, recent advances in TRS remodeling, such as altering core sequences and flanking contexts in PEDV, have demonstrated that shifting the sgRNA abundance hierarchy can attenuate viral replication while boosting host interferon responses, offering a novel rationale for designing recombination-resistant live attenuated vaccines [83, 84], which was consistent with the data obtained with human SARS-CoV-1 TRS mutants [82]. Given that TRSs play critical roles in viral recombination and mediate viral transcription, alterations to the core consensus sequences of TRS may reduce the fitness or viability of recombinants by imposing transcriptional incompatibility because TRS-core rewiring may impose transcriptional incompatibility on recombinants [82-84]. Consequently, live attenuated vaccines incorporating mutations within the TRS core sequences may represent a promising modality for vaccine safety, while simultaneously conferring immunological protection through conventional vaccination mechanisms (Fig. 2) [85-87]. A recent study demonstrated that SARS-CoV-2 cDNA can be engineered as a live attenuated vaccine (LAV) platform capable of expressing heterologous antigens, using Omicron JN.1-based viral backbones combined with targeted modifications including mutations in the viral nsp16 protein (D130A), deletion of the furin cleavage site in spike, deletion of accessory proteins, and modification of transcription regulatory sequences. When expressing a nonfunctional, stabilized prefusion form of respiratory syncytial virus F protein (RSVF), the optimized construct (rJN.1-D130A-dFCS-RSVF) remained sufficiently attenuated while being highly immunogenic, providing complete protection against challenge with both JN.1 and RSV in hamster models [88]. It is important to note that coronaviruses with completely rewired transcription regulatory networks (TRNs) can serve as effective vaccine platforms. Small changes to TRS sites, as few as 3 nucleotides, revert through compensatory mutations during serial passage, allowing the transcriptional program to return to wild-type and phenotypic reversion to occur. More extensive TRS modifications, particularly 7-nucleotide changes, are genetically stable through serial passage. This suggests that sufficiently extensive rewiring of the transcriptional regulatory network is necessary to prevent compensatory evolution and maintain attenuation in live attenuated vaccine candidates [85]. In fact, the capacity of coronaviruses to serve as vectors is fundamentally enabled by their transcription regulatory sequences (TRSs), which permit systematic evaluation of foreign gene expression at different genomic positions. This modular design allows heterologous genes to be inserted with viral transcription signals into the genome and efficiently expressed through the coronavirus discontinuous transcription mechanism [89, 90]. Moreover, the location of the core TRS sequence relative to its flanking nucleotides substantially influences transcription efficiency. Variations in the flanking region, whether natural variants from other viral genes or experimental substitutions, can shift sgRNA abundance by 10- to 20-fold, or in extreme cases eliminate the junction entirely (Fig. 3A). This shows that a TRS is not fully defined by its core motif alone; the surrounding sequence context is part of the functional element. This context-dependency necessitates careful optimization of TRS positioning, as even a highly conserved core sequence at an inappropriate genomic locus may fail to generate productive mRNA synthesis [24, 52].

The use of coronaviruses as vaccine vectors builds upon established principles from other viral platforms. Several features make coronaviruses attractive as vaccine and therapeutic vectors: deletion of nonessential genes is strongly attenuating; the genetic space thus created allows insertion of foreign genetic information; and tropism can be modified through manipulation of the viral spike protein [89]. While the recent SARS-CoV-2/RSV work represents a novel application of SARS-CoV-2 specifically, the broader strategy of using coronavirus genomes as carrier platforms has precedent. Specifically, coronavirus-derived minigenomes, self-replicating RNA replicons lacking structural protein genes, can accommodate large foreign inserts, making them attractive vaccine platforms. Because the replicon replicates efficiently yet produces no infectious particles on its own, it is inherently safer than working with replication-competent virus. Moreover, detailed characterization of TRS function through helper-virus-dependent systems has enabled rational design of the transcriptional regulatory elements in minigenome vaccines, allowing researchers to optimize which genes are expressed and at what levels. This combination of large cloning capacity, built-in safety, and predictable control over gene expression suggests that engineered coronavirus vectors could be developed as vaccines against multiple pathogens [52]. Further investigations into this novel approach may elucidate its potential clinical applications and efficacy [91].

### Open Questions and Future Directions

Several fundamental questions remain unresolved. First, can TRS sequence alone predict sgRNA abundance, or are RNA structure and long-range interactions necessary? Available data suggest the latter, but rigorous testing requires targeted mutations and integrated computational models [5, 14, 25-29]. Second, what molecular mechanisms explain the patterns of junctions observed in transcriptome maps? Answering this requires incorporating both RTC kinetics and RNA conformational dynamics into current models, which neither has yet integrated [5, 24, 25].

Third, how do we connect structural snapshots of the RTC, static images from cryo-EM or ensemble data, to the dynamic decisions the polymerase makes during pause and switching? This requires time-resolved and single-molecule approaches that can track polymerase behavior in real time [24, 31-33]. Fourth, how do the rules governing discontinuous transcription relate to recombination? Both depend on template switching, so understanding how switching efficiency is controlled could explain both the short-term regulation of gene expression and the long-term evolutionary diversity of coronaviruses [4, 9, 73-76]. Fifth, do different coronavirus lineages use the same transcription principles, or has each evolved its own switching rules? Comparing TRS sequences and transcription patterns across the coronavirus family could identify conserved design principles versus lineage-specific innovations [4, 9, 14]. Sixth, how does the protein environment shape TRS selection? The nucleocapsid protein is an obvious candidate, but distinguishing a general chaperone activity from site-specific recognition, and either from the effects of compartmentalization, will require conditional perturbation of N during ongoing transcription rather than comparison of N mutants at the level of bulk sgRNA yield. Finally, how do viral transcription programs interact with host defenses? Integrating TRS biology with knowledge of host factors and innate immune recognition could reveal how the virus times gene expression to evade immune detection, and may suggest new targets for intervention [79, 92].

## Conclusion

TRSs are central RNA regulatory signals that translate sequence and structural context into discontinuous transcription outcomes, thereby defining the coronavirus gene-expression program [4, 5, 9, 14]. Transcriptome mapping has shown that canonical sgRNAs coexist with extensive non-canonical junction diversity, establishing a probabilistic switching process sensitive to RNA context and RTC behavior [5, 21, 22]. Genome-wide RNA structure and interactome studies demonstrate that TRS function cannot be reduced to a core motif alone but emerges from multi-scale RNA architectures that shape accessibility and kinetics of leader–body pairing [14, 25-29, 63].

The RTC, now characterized structurally as a multi-protein assembly, provides the enzymatic machinery that executes pausing and switching decisions in response to TRS signals [24, 31-33, 80, 93]. Linkages between discontinuous transcription and recombination underscore that TRS-mediated template switching is not only a gene-expression mechanism but also a driver of genome evolution and antigenic variation [4, 9, 73-76], and the rewiring of such mechanisms might provide safe and effective ways, thereby reducing the risk of recombination between circulating field strains and vaccine strains. Moreover, continued integration of structural biology, high-resolution transcriptomics, and *in vivo* RNA structural measurements should enable predictive models and, ultimately, new strategies to modulate or disrupt TRS-mediated transcription for translational applications.

## Figures and Tables

**Fig. 1:**
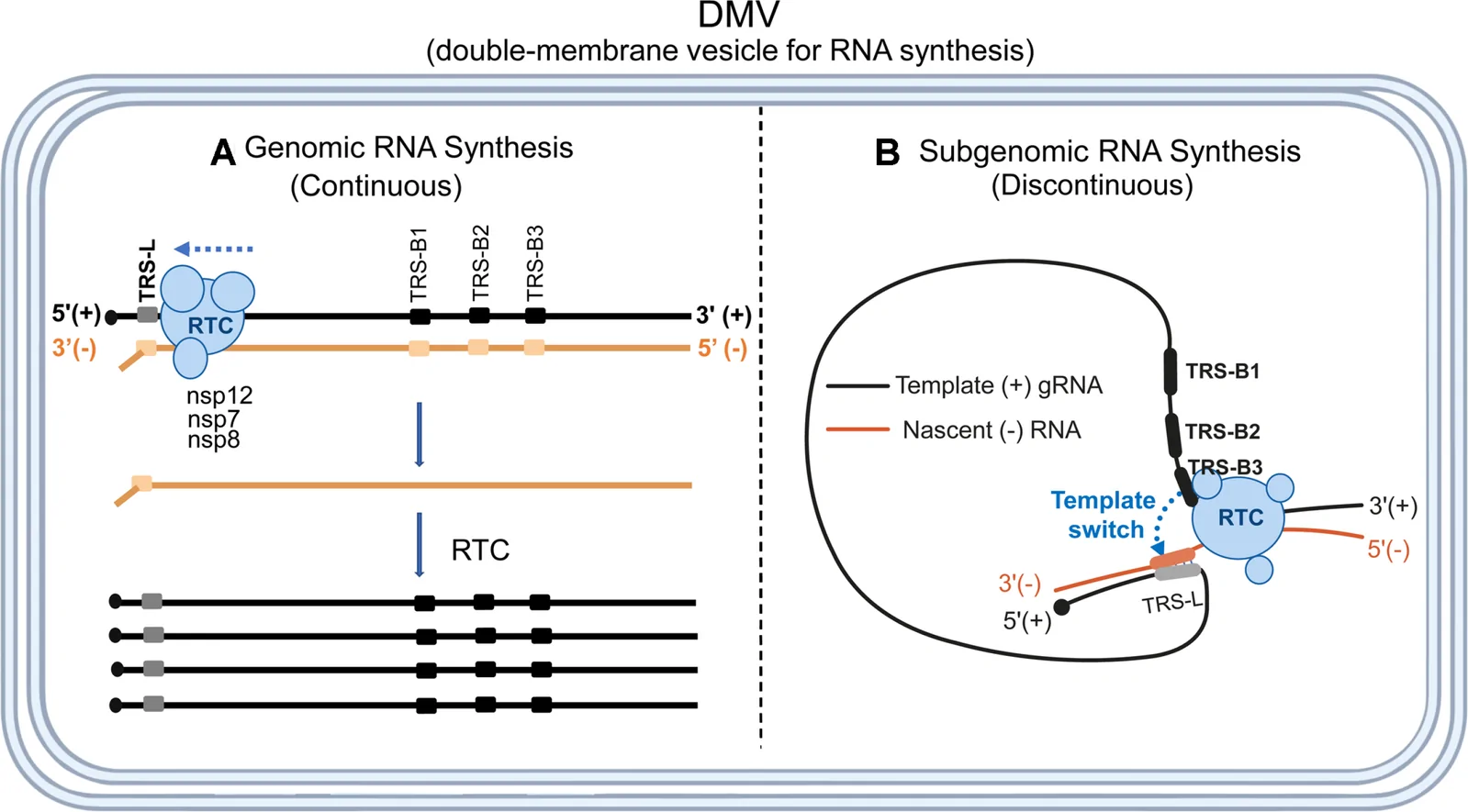
Two modes of coronavirus RNA synthesis within the double-membrane vesicle (DMV). (**A**) Continuous genomic RNA synthesis. The RTC, composed of nsp12 (RdRp) together with its cofactors nsp7 and nsp8, binds the 3′ end of the positive-sense genomic RNA (gRNA) and translocates along the template to synthesize a complementary negative-sense strand. The blue dashed arrow indicates the direction of RTC movement along the template. In this mode the RTC reads through all body transcription-regulatory sequences (TRS-B1–B3) as well as the leader TRS (TRS-L), producing a full-length (−) strand RNA. This full-length (−) strand then serves as a template for the amplification of multiple progeny (+) strand genomes. (**B**) Discontinuous subgenomic RNA synthesis. The same RTC initiates (−) strand synthesis from the 3′ end of the (+) gRNA but pauses upon reaching a body TRS (TRS-B3, B2, or B1). The anti-TRS at the 3′ end of the nascent (−) strand then base-pairs with the TRS-L located in the 5′ leader region of the genome, mediating a template switch; the blue dashed arrow indicates the direction of RTC movement as it relocates from the TRS-B to the TRS-L. Synthesis resumes on the leader sequence, generating a leader-fused negative-sense subgenomic RNA (sgRNA). Template switching at different TRS-B sites yields a nested set of sgRNAs of distinct lengths, which subsequently serve as templates for the corresponding (+) strand sgRNAs. Black lines denote the template (+) gRNA; orange lines denote nascent (−) strand RNA. Gray boxes indicate TRS-L, black boxes indicate body TRSs (TRS-B1–B3), and orange boxes indicate the complementary anti-TRS elements generated on the nascent (−) strand. DMV, double-membrane vesicle; RdRp, RNA-dependent RNA polymerase; RTC, replication–transcription complex; TRS, transcription-regulatory sequence.

**Fig. 2:**
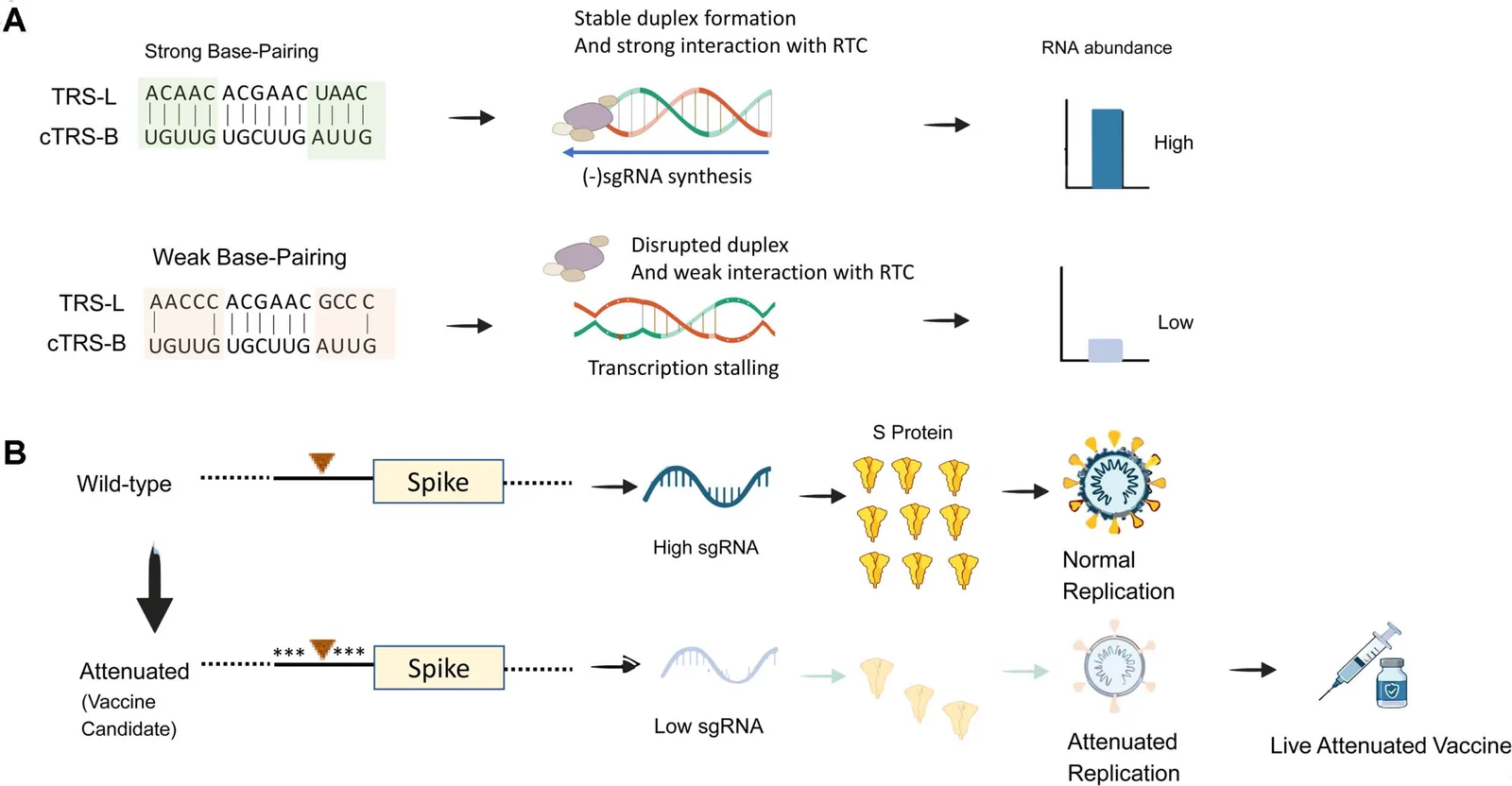
Disruption of transcription regulatory sequence (TRS) duplex stability reduces coronavirus subgenomic RNA transcription and enables rational design of live-attenuated vaccines. (**A**) Mechanism of transcription attenuation via TRS mutagenesis. Strong base-pairing complementarity between the leader TRS (TRS-L) and the complementary body TRS (cTRS-B) promotes stable RNA duplex formation and robust interaction with the replication-transcription complex (RTC), resulting in efficient negative-sense subgenomic RNA ((-)sgRNA) synthesis and high viral RNA abundance (top). Introducing mutagenesis into the flanking sequences surrounding the TRS core sequence disrupts base-pairing complementarity, leading to duplex destabilization, transcription stalling, and weak association with the RTC, which significantly suppresses overall RNA abundance (bottom). (**B**) Strategy for developing a rationally engineered live-attenuated virus vaccine candidate. In wild-type coronavirus, intact TRS duplex formation drives high expression of subgenomic RNA encoding structural proteins (*e.g.*, Spike protein), sustaining normal viral replication (top). Introducing targeted point mutations (represented by asterisks, ***) adjacent to the TRS region (brown triangle) attenuates sgRNA synthesis, markedly reducing downstream viral protein expression and yielding viral replication attenuation without compromising genomic integrity—forming the basis of a safe, live-attenuated vaccine candidate (bottom).

**Fig. 3:**
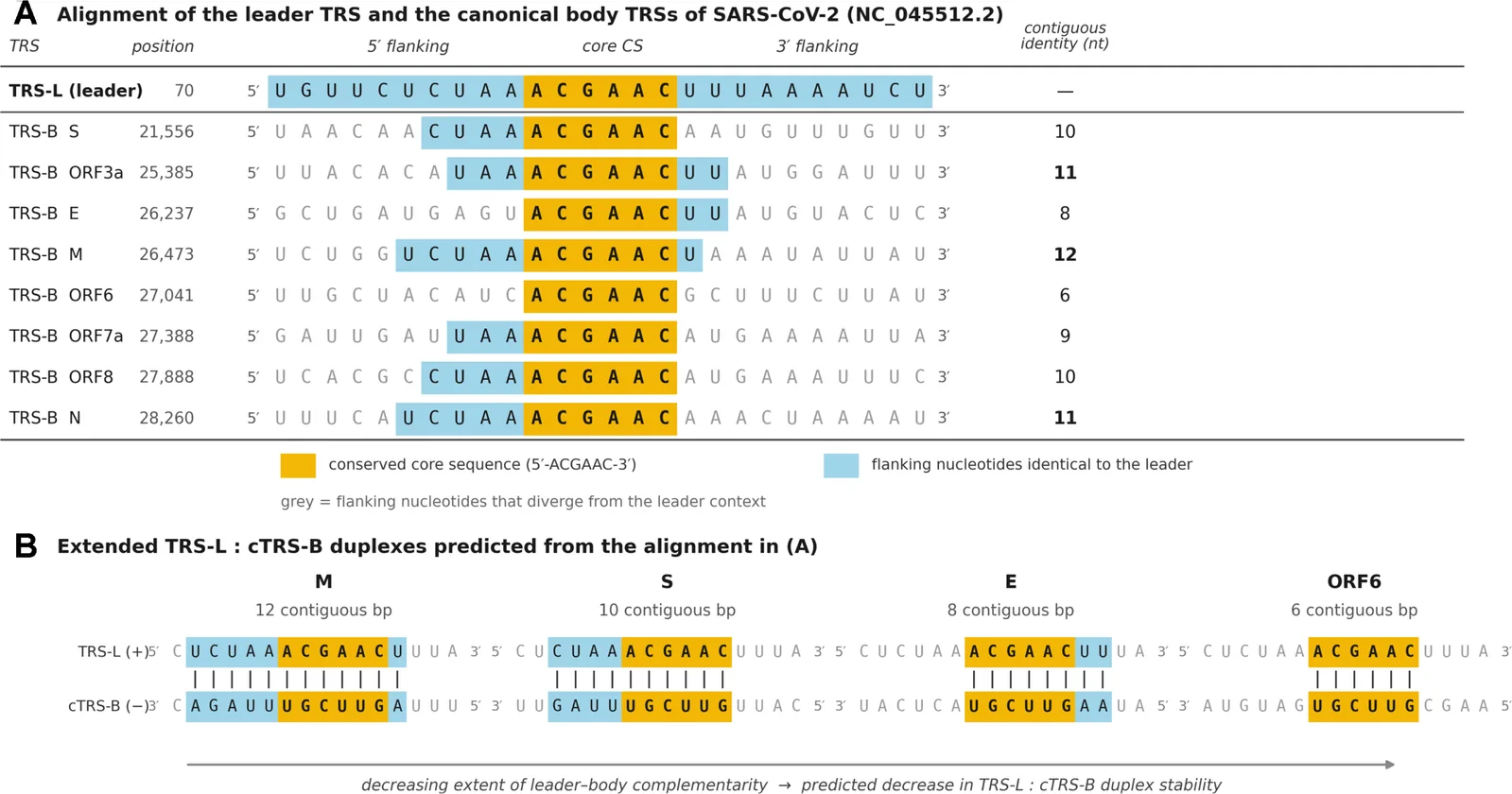
Sequence relationships between the leader TRS and the canonical body TRSs of SARS-CoV-2. (**A**) Alignment of the leader TRS (TRS-L, genome position ~70) with the nine canonical body TRSs (TRS-B) of the SARS-CoV-2 reference genome (NC_045512.2). For each TRS, the genomic position is indicated at left, and the sequence is shown in the 5′→3′ orientation with the 5′ flanking region, the core sequence (CS), and the 3′ flanking region aligned on the CS. The conserved core sequence (5′-ACGAAC-3′) is shaded in orange; flanking nucleotides identical to the corresponding positions of the leader are shaded in blue; flanking nucleotides that diverge from the leader context are shown in grey. The right-hand column gives the contiguous identity to TRS-L, defined as the length (in nucleotides) of the longest uninterrupted stretch of identity spanning the core CS; that is, the 6-nt core plus the flanking nucleotides on either side that match the leader without any mismatch; counting stops at the first mismatch on each side, and matching nucleotides lying beyond that mismatch are not included. Contiguous identity two fold among the canonical TRS-B sites, from 12 nt (**M**) and 11 nt (ORF3a, N) down to 6 nt (ORF6), despite the invariant core CS. (**B**) Extended TRS-L : cTRS-B duplexes predicted from the alignment in (**A**), shown for four representative body TRSs (M, S, E and ORF6) spanning the observed range of complementarity. In each pair, the plus-strand leader TRS (TRS-L (+), top, 5′→3′) is base-paired with the complement of the nascent minus-strand body TRS (cTRS-B (−), bottom, 3′→5′); vertical lines denote Watson–Crick base pairs. Shading is as in (**A**), with the core CS/complement in orange and leader-identical flanking pairs in blue. The predicted duplex extends well beyond the 6-bp core wherever flanking identity is retained, giving 12, 10, 8 and 6 contiguous base pairs for M, S, E and ORF6, respectively. The arrow below indicates the resulting gradient: decreasing leader–body complementarity is expected to translate into progressively lower TRS-L : cTRS-B duplex stability, and hence into differing efficiencies of template switching during discontinuous negative-strand synthesis.

**Table 1:** Continuous vs Discontinuous Transcription.

	Continuous transcription	Discontinuous transcription
RdRp at a body TRS	Reads through	Attenuates, then switches template
Template	Whole (+)-gRNA is copied	Region between TRS-B and TRS-L is bypassed
Immediate product	Full-length (-)-gRNA	Shorter (-)-sgRNA
Final product	Progeny (+)-gRNA	Capped, subgenomic mRNA
Leader-body junction	Absent	Present (TRS-L fused to the TRS-B)
Role in infection	Genome amplification and packaging	Expression of downstream ORFs

**Table 2:** Comparative features of the 5′-terminal stem-loop 3 (SL3) region and the presentation of TRS-L across representative coronaviruses.

Virus	SL3	Position of TRS-L	Evidence	Functional implication
SARS-CoV	Stable hairpin	3′ half of the SL3 stem	NMR [46]	TRS-L partially base-paired; release required before pairing with cTRS-B
SARS-CoV-2	No single dominant fold; folded ↔ partially open ensemble	3′ side of the stem in the folded state	Genome-wide probing and comparative/computational analyses	Transient exposure of TRS-L; conformational equilibrium may set switching probability
BCoV	Hairpin present	Hairpin loop	Comparative structure analysis	TRS-L predominantly loop-exposed
MHV	SL3 unstable at 37°C	Stem/loop boundary	Thermodynamic and structural modelling	Rapid conformational transitions; low barrier to TRS-L exposure
MERS-CoV	Absent	Single-stranded	Comparative analysis of 5′-terminal *cis*-acting elements	Structure-independent TRS-L presentation; alternative regulation
